# Respiratory Diseases

**Published:** 1903-11-14

**Authors:** 


### RESPIRATORY DISEASES.

*Concluded from page* 103.

**Human and Bovine Tuberculosis.**—Nathan Raw[14] adopts the view that there are two distinct diseases. The bovine form is much more virulent, is capable of infecting man, and is very prone to attack children. He considers that the forms of tuberculosis that children are prone to are usually a bovine infection, while the adult phthisical type is usually of human origin. The typhoid form of general tuberculosis is perhaps bovine. He throws out the valuable hint, that, since human tubercle cannot be implanted on cattle already suffering from tuberculosis perhaps the two forms cannot co-exist in man, and the serum of a tuberculous cow might prove to be curative in cases of phthisis in the same way that cow-pox and small-pox are antagonistic. Kober[15] has tabulated 86 cases of tuberculosis, in which he considers that the infection was milk-borne bovine tuberculosis. Carmichael has shown the proneness of children to infection by milk after weaning in 49 cases. Behring[16] hopes to make use of "anti" bodies present in the milk of tuberculous cows to immunise human beings. His animals, such as survived inoculation with attenuated cultures of tubercle bacilli from human sources, showed their reaction by fever, with pneumonic consolidation and some pleural effusion which cleared up in three or four weeks, and their serum then had a very strong agglutinating power. They were then immune, and could resist infection under the most unfavourable conditions. Suspensions of tubercle bacilli can now be made so that the agglutination reaction can be of practical use, and we are able to test whether patients who are receiving a tubercle vaccine are reacting properly. The difficulty in previous attempts at vaccination has been that the patient's resistance is at first lowered (the negative phase) as in other kinds of vaccination, diphtheria for example in horses, and if the second inoculation is made during this negative phase, the resistance of the subject is still further decreased. If, however, a sufficient interval be allowed to elapse for the positive phase of increased resistance to follow, a further inoculation produces first its own negative phase, and then a still higher degree of resisting power. This is what is now done, according to Wright[17], in Koch's clinic. In this paper, Wright puts forward a theory to account for the agglutination reactions, bringing the process into line with the sedimentation of river deposits by sea-water and the gelatinisation of precipitates by saline solutions.

**Pneumonia.**—Several observers[18] report on the use of pilocarpine in cases of pneumonia. Its action is said to be mechanical, producing free bronchial secretion which removes the organisms from the tubes. Coughing is facilitated and is attended with less pain, due, it is supposed, to slight mechanical out-pouring of fluid on to the pleural surfaces, the result of hyperæmia. The drug, though it produces some diaphoresis, has little or no action on the fever nor on the toxæmic condition, but the crisis appears to be brought about sooner, and resolution is accelerated. The liability to spread to other lobes is not, however, lessened. A single dose is given, of a tenth of a grain. Smaller repeated doses tend to produce cardiac depression, and have less influence on the disease. Mongour[19] reports a case of pneumothorax resulting from an exploratory puncture. Death from asphyxia appeared imminent, but the physician saved his case from immediate death by introducing a large cannula at the site of the puncture. The pent-up air escaped with a whistle, and the patient was at once relieved of dyspnœa.

**A New Form of Aspirator,**[20] one which is very much simpler and cheaper than the arrangement of stopcocks and exhausting pump commonly in use, is employed at the New York Hospital. It consists of a large bottle, such as a "Winchester," with a rubber stopper through which a tube is introduced, connecting it with the aspirator needle. A partial vacuum is produced by burning the vapour from two or three drachms of 95 per cent. alcohol shaken up in the bottle. When the flame has very nearly died out the stopper is inserted. The connecting tube should be of stout rubber, and the flow through it may be regulated by a tap or clamp, or pair of Spencer-Wells forceps. Fifty per cent. alcohol (whiskey, etc.) will serve to produce the vacuum if the bottle be previously warmed up to 85° F., but will not burn at ordinary temperatures, while strong alcohol, on the other hand, is liable to explode if the temperature of the bottle is raised above ordinary room heat. The products of combustion must be displaced by filling the bottle with water before it can be used a second time.

Bronchitis Fibrinosa.—Hockhaus[21] contributes to the pathology of this rare disease a report of a case in which he found that the casts were composed of mucin, with a few epithelial cells and leucocytes caught up in the mass. In some cases the casts consist of fibrin, but lately examination has shown that there are instances like the present one in which they consist of mucin.

Gold Miners' Phthisis.[22]—The Transvaal authorities have appointed a commission to report on the health among workers in the gold mines of the colony, and the Chamber of Mines has offered prizes for suggestions as to the best means of obviating the inhalation of the fine dust produced by the rock-drills and blasting. The disease is probably due to the fine dust in the inhaled air, and is one of the pneumoconioses, though possibly the irritating fumes of the blasting powders have a share in its production. In this country good ventilation of the mines has made coal mining one of the healthiest of our occupations Clifford Allbutt,[23] speaking at Glasgow on "Tuberculosis," compared the tubercle bacillus to the flogging schoolmaster of the first half of the last century, not exactly a friend, but a benefactor. The man of common sense is being scourged into recognising the teaching of the laboratory worker; the minute details which excited his derision are now by their overwhelming number conquering or rather coinciding with facts to which at first they appeared antagonistic. The speaker insists on the infectiousness of tuberculosis, but raises a protest against the idea that the profession is attaching too much importance to the bacillus and not enough to the human "garden." It is, in fact, the garden that has received perhaps an undue share of attention, more might well be given to the bacillus. And to compare the human body to "a soil, that is a medium more or less proper and conducive to the germination of seed," is misleading. The study of the pathological reactions in this and other infections has completely changed our views as to our conduct in regard to them. We must avoid infection, but it is more important that we should cultivate the "soil," and the work of the sanatorium will be of value in educating the community to understand the virtues of fresh air, and how they may be utilised in spite even of low temperature and absence of sunlight. The fear that by this work we shall propagate "bad stocks," is unfounded. By our ignorance, and by the ignorance of our fathers, we have weakened the child. Perhaps we provide it with a tuberculous infection during the time when it "inhabits the floor," which breaks into activity later on. We should blame the gardeners for the production of bad stock; when we can be sure that our gardeners are perfect, we can then set about its elimination.

[14] Lancet, March 7, and Brit. Med. Jour., March 14. [15] Med. Rec., May 23. [16] Brit. Med. Jour., April 4. [17] Lancet, May 9. [18] Lancet, Mar. 21 and May 16. [19] Lancet, July 11. [20] Med. Rec., July 4. [21] Quar. Med. Jour., Feb. [22] Lancet, July 4. [23] Practitioner, Feb.

